# Medical education in the midst of the COVID-19 pandemic: The challenge of collaborative learning in three european countries

**DOI:** 10.1192/j.eurpsy.2021.1593

**Published:** 2021-08-13

**Authors:** D. Gurrea Salas, R. Palma Álvarez, S.M. Toparlak

**Affiliations:** 1 Mental Health Center, Klinikum stuttgart, stuttgart, Germany; 2 Psychiatry, Hospital Universitari Vall d’Hebron, Barcelona, Spain

**Keywords:** training programs, E-Learning, COVID-19, collaborative learning

## Abstract

**Introduction:**

COVID19 keeps being a challenge, not only facing the outbreak and the treatment of the cases, but also in the education sector. Most learning centres and high schools in the world are closed to avoid further outbreaks, as well as institutes for psychotherapy throughout the world.

**Objectives:**

To gain a better knowledge and understanding about alternatives identified in the scope of psychiatric trainee training, through the support provided by digital resources.

**Methods:**

Systematic review on PubMed and Uptodate databases since declaration of the COVID-19 pandemic in March 2020 was performed using the keywords: Distance Education, Pandemia, COVID-19, Medical Residency. Discussing online-learning.

**Results:**

The described European countries (Germany, Spain, United Kingdom) used different strategies to maintain the e-learning. Practical undergraduate education was replaced in countries like Spain by “problem-based learning” tasks, clarifying and commenting case reports or videos through working groups. The increase of the resources from teachers and trainers wasn´t taken in account for the preparation of the digital program. Social inequities for the digital access for groups of students or clients were also claimed.

**Conclusions:**

Each of the described countries adopted different strategies regarding continuing training of residents, their assessment and their certification. Covid-19 should set down a trend of social collaborative learning as part of resident training and asset hybrid or even digital methods for the mental health training.

